# ﻿*Mordellistenaplatypoda*, a new species of tumbling flower beetle from the island of Ischia in Italy (Coleoptera, Mordellidae)

**DOI:** 10.3897/zookeys.1148.86845

**Published:** 2023-02-14

**Authors:** Dávid Selnekovič, Katarína Goffová, Ján Šoltýs, Eva Kováčová, Ján Kodada

**Affiliations:** 1 Department of Zoology, Comenius University Bratislava, Faculty of Natural Sciences, Ilkovičova 6, 842 15, Bratislava, Slovakia Comenius University Bratislava Bratislava Slovakia; 2 Institute of Electrical Engineering, Slovak Academy of Sciences, Dúbravská cesta 9, 841 04, Bratislava, Slovakia Institute of Electrical Engineering, Slovak Academy of Sciences Bratislava Slovakia

**Keywords:** Cytochrome c oxidase subunit I (COI), DNA barcoding, integrative taxonomy, morphology, species delimitation

## Abstract

*Mordellistena* A. Costa, 1854, the most species-rich genus of tumbling flower beetles comprises more than 800 species worldwide and more than 150 reported from Europe. Here, a new species Mordellistena(s. str.)platypoda is described from the island of Ischia in Italy. The species hypothesis is based primarily on morphological characters which are visualised using scanning electron microscopy images, high-resolution photographs, and drawings. The species hypothesis is supported by analysis of a 658 bp fragment of cytochrome c oxidase subunit I (COI). Divergences in the COI gene are evaluated using maximum likelihood and Bayesian inference analyses. The species delimitation is assessed using Assemble Species by Automatic Partitioning (ASAP) and Poisson Tree Processes (PTP) methods. Genetic distances are visualised using multidimensional scaling. *Mordellistenaplatypoda* Selnekovič, Goffová & Kodada, **sp. nov.** is recovered as a well-separated species by both molecular and morphological analyses. Our results show that *M.platypoda* Selnekovič, Goffová & Kodada, **sp. nov.** is most closely related to *M.tarsata* Mulsant, 1856, although the two species differ significantly in vestiture colouration, presence of lateral ctenidia on the third metatarsomere, and presence of sexual dimorphism on the protibia. The results indicate that such morphological differences, which were traditionally used to distinguish between species groups, may in fact be present between closely related species. Interestingly, examination of the numerous museum material did not reveal additional specimens of the new species, and therefore *M.platypoda* Selnekovič, Goffová & Kodada, **sp. nov.** is currently known only from the Italian island of Ischia.

## ﻿Introduction

More than 800 species are currently classified within *Mordellistena* A. Costa, 1854, making it the most species-rich genus of tumbling flower beetles. It is reported from every continent except Antarctica. However, the generic placement of many species, especially Indomalayan and Neotropical, needs to be reassessed according to the current generic classification. In Europe, the genus is represented by more than 150 species (Horák 2020), which are pollinivorous in the adult stage and are among the most frequently encountered flower-visiting beetles. Larval stages are documented in approximately 18 European species (ca. 12%) and develop in the stems of herbaceous plants, feeding on the plant tissue (e.g., Borowiec 1996; Odnosum 2010; Zemoglyadchuk and Buialska 2020). The genus was generally characterised in terms of morphology by Ermisch (1950) and Franciscolo (1957). Identification of the European species is based on the concept of the species groups defined by combinations of morphological characters (Ermisch 1956, 1977). Due to the homogeneity in the external morphology, species identification is often challenging and is possible only by examination of the genitalia and comparison with the type specimens. Essential works covering the European representatives of the genus, including identification keys and figures of diagnostic characters, were provided by Costa (1854), Mulsant (1856), Schilsky (1894, 1895, 1898), Ermisch (1956, 1963, 1969, 1977), Batten (1977), and Horák (1983, 1990, 1996). The comprehensive catalogue of Palaearctic fauna was provided by Horák (2008, 2020). The phylogeny of the genus has not yet been studied.

**Figure 1.**
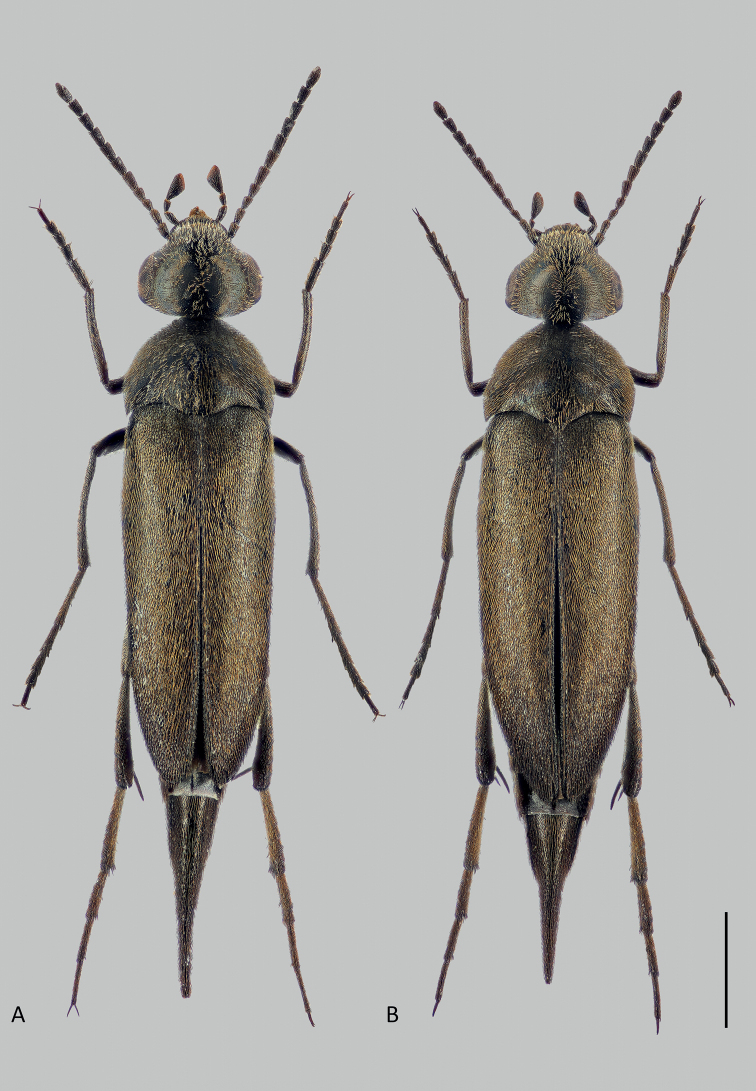
*Mordellistenaplatypoda* Selnekovič, Goffová & Kodada, sp. nov. **A** male paratype **B** female paratype. Scale bar: 1.0 mm.

A recent collecting trip to the island of Ischia in Italy in June 2019 yielded more than 1,000 Mordellidae specimens representing 12 species. Within this material, we recognised a series of 52 specimens belonging to the new species described herein, *Mordellistenaplatypoda* Selnekovič, Goffová & Kodada, sp. nov. (Fig. 1). The aim of the present study was to describe the new species and delimit the species boundaries. To achieve this goal through an integrative approach, we tested the morphology-based species hypothesis with the results of single-gene DNA analyses. We documented the morphological characters using scanning electron microscopy, high-resolution photographs, and line drawings, and compared the new species with morphologically similar congeners. In order to analyse divergences in the mitochondrial gene that encodes cytochrome c oxidase subunit I (COI), we applied two probabilistic statistical methods (maximum likelihood and Bayesian inference) and compared the results with distance-based (Assemble Species by Automatic Partitioning) and tree-based (Poison Tree Processes) species delimitation analyses.

## ﻿Materials and methods

This study is based on the examination of more than 400 specimens of the genus *Mordellistena* deposited at the following institutions:

**HNHM**Magyar Természettudományi Múzeum, Budapest, Hungary;

**MNHU** Museum für Naturkunde der Humboldt Universität, Berlin, Germany;

**MSNG**Museo di Storia Naturale Giacomo Doria, Genova, Italy;

**MZFN**Museo Zoologico dell’Università Federico II, Naples, Italy;

**SDEI**Senckenberg Deutsches Entomologisches Institut, Müncheberg, Germany;

**SNSD** Senckenberg Naturhistorische Sammlungen, Dresden, Germany.

The type series of *M.platypoda* consist of 52 specimens collected on the island of Ischia in Italy. The specimens of the additional species included in the phylogenetic analyses were acquired from several localities in Italy, Slovakia, Spain, Cyprus, and Bulgaria (Table 1). Type specimens of *M.platypoda* were compared with those of the following morphologically similar species: *M.austriaca* Schilsky, 1898 (male lectotype and two paralectotypes, MNHU), *M.balianii* Franciscolo, 1942 (male holotype and two paratypes, MSNG), *M.hirtipes* Schilsky, 1895 (male lectotype and 35 paralectotypes, MNHU), *M.micans* (Germar, 1817) (two female syntypes, SDEI), *M.pseudohirtipes* Ermisch, 1965 (male holotype and one paratype, SNSD), *M.purpurascens* A. Costa, 1854 (male lectotype, MZFN), and *M.tenuicornis* Schilsky, 1898 (male lectotype and 17 paralectotypes, MNHU).

**Table 1.** List of specimens used in DNA analyses.

Specimen ID	GenBank	Locality	Coordinates
*Mordellistenaaustriaca* DSBS 60	OM680976	Slovakia, Virt env.	47.760000°N, 18.340556°E
*Mordellistenaconfinis* DSBS 243	OP586774	Italy, Firenze, Cinipetta	43.570000°N, 11.420556°E
*Mordellistenaconfinis* DSBS 244	OP586776	Italy, Firenze, Cinipetta	43.570000°N, 11.420556°E
*Mordellistenaconfinis* DSBS 245	OP586769	Italy, Firenze, Cinipetta	43.570000°N, 11.420556°E
*Mordellistenaconfinis* DSBS 250	OP586770	Italy, Firenze, Cinipetta	43.570000°N, 11.420556°E
*Mordellistenaconfinis* DSBS 329	OP586775	Italy, Firenze, Cinipetta	43.570000°N, 11.420556°E
*Mordellistenahirtipes* DSBS 205	OM681007	Cyprus, Limassol env.	34.755278°N, 33.093333°E
*Mordellistenahirtipes* DSBS 207	OM681008	Cyprus, Limassol env.	34.755278°N, 33.093333°E
*Mordellistenahirtipes* DSBS 208	OM681009	Cyprus, Limassol env.	34.755278°N, 33.093333°E
*Mordellistenalindbergi* DSBS 144	OM680979	Cyprus, Limassol env.	34.755278°N, 33.093333°E
*Mordellistenalindbergi* DSBS 270	OP586772	Cyprus, Akamas	35.057586°N, 32.345527°E
*Mordellistenalindbergi* DSBS 271	OP586767	Cyprus, Akamas	35.057586°N, 32.345527°E
*Mordellistenalindbergi* DSBS 280	OP586771	Cyprus, Foinikaria env.	34.766272°N, 33.100258°E
*Mordellistenaminima* DSBS 79	MT232550	Italy, Ischia, Serrara env.	40.716667°N, 13.886389°E
*Mordellistenaminima* DSBS 172	OM680982	Italy, Ischia, Serrara env.	40.716667°N, 13.886389°E
*Mordellistenaminima* DSBS 173	OM680983	Italy, Ischia, Serrara env.	40.716667°N, 13.886389°E
*Mordellistenaminima* DSBS 215	OP586765	Italy, Sardinia, Castiadas	39.206812°N, 9.562685°E
*Mordellistenaminima* DSBS 218	OP586768	Italy, Sardinia, Castiadas	39.206812°N, 9.562685°E
*Mordellistenaminima* DSBS 219	OP586766	Italy, Sardinia, Castiadas	39.206812°N, 9.562685°E
*Mordellistenaplatypoda* DSBS 83	OM680977	Italy, Ischia, Serrara env.	40.716667°N, 13.886389°E
*Mordellistenaplatypoda* DSBS 118	OM680978	Italy, Ischia, Serrara env.	40.716667°N, 13.886389°E
*Mordellistenaplatypoda* DSBS 194	OM680997	Italy, Ischia, Serrara env.	40.716667°N, 13.886389°E
*Mordellistenaplatypoda* DSBS 195	OM680998	Italy, Ischia, Serrara env.	40.716667°N, 13.886389°E
*Mordellistenaplatypoda* DSBS 199	OM681002	Italy, Ischia, Serrara env.	40.716667°N, 13.886389°E
*Mordellistenaplatypoda* DSBS 233	OM681019	Italy, Ischia, Serrara env.	40.716667°N, 13.886389°E
*Mordellistenaplatypoda* DSBS 235	OM681020	Italy, Ischia, Serrara env.	40.716667°N, 13.886389°E
*Mordellistenaplatypoda* DSBS 237	OM681021	Italy, Ischia, Serrara env.	40.716667°N, 13.886389°E
*Mordellistenapseudorhenana* DSBS 11	OP586773	Cyprus, Foinikaria env.	34.755278°N, 33.093333°E
*Mordellistenapseudorhenana* DSBS 12	MT232533	Cyprus, Foinikaria env.	34.755278°N, 33.093333°E
*Mordellistenapseudorhenana* DSBS 43	MT232539	Slovakia, Chotín env.	47.806389°N, 18.198056°E
*Mordellistenapurpurascens* DSBS 82	MT232552	Italy, Ischia, Serrara env.	40.721389°N, 13.883056°E
*Mordellistenapurpurascens* DSBS 111	MT232554	Spain, Tossa de Mar	41.736667°N, 2.935000°W
*Mordellistenapurpurascens* DSBS 117	MT232555	Italy, Ischia, Serrara env.	40.721389°N, 13.883056°E
*Mordellistenapurpurascens* DSBS 182	OM680985	Italy, Ischia, Serrara env.	40.716667°N, 13.886389°E
*Mordellistenapurpurascens* DSBS 183	OM680986	Italy, Ischia, Serrara env.	40.716667°N, 13.886389°E
*Mordellistenapurpurascens* DSBS 186	OM680989	Italy, Ischia, Serrara env.	40.716667°N, 13.886389°E
*Mordellistenapurpurascens* DSBS 187	OM680990	Italy, Ischia, Serrara env.	40.716667°N, 13.886389°E
*Mordellistenapurpurascens* DSBS 188	OM680991	Italy, Ischia, Serrara env.	40.716667°N, 13.886389°E
*Mordellistenapurpurascens* DSBS 192	OM680995	Italy, Ischia, Serrara env.	40.716667°N, 13.886389°E
*Mordellistenapurpurascens* DSBS 200	OM681003	Italy, Ischia, Serrara env.	40.716667°N, 13.886389°E
*Mordellistenapurpurascens* DSBS 201	OM681004	Italy, Ischia, Serrara env.	40.716667°N, 13.886389°E
*Mordellistenapurpurascens* DSBS 226	OM681016	Italy, Sardinia, Castiadas env.	39.206812°N, 9.562685°E
*Mordellistenapurpurascens* DSBS 227	OM681017	Italy, Sardinia, Castiadas env.	39.206812°N, 9.562685°E
*Mordellistenapurpurascens* DSBS 231	OM681018	Italy, Ischia, Serrara env.	40.716667°N, 13.886389°E
*Mordellistenapurpureonigrans* DSBS 49	OP575375	Slovakia, Chotín env.	47.806389°N, 18.198056°E
*Mordellistenapurpureonigrans* DSBS 55	OM680974	Slovakia, Virt env.	47.760000°N, 18.340556°E
*Mordellistenapurpureonigrans* DSBS 56	OM680975	Slovakia, Virt env.	47.760000°N, 18.340556°E
*Mordellistenapurpureonigrans* DSBS 71	OP575374	Slovakia, Iža env.	47.748056°N, 18.260556°E
*Mordellistenapurpureonigrans* DSBS 73	OP575376	Slovakia, Iža env.	47.748056°N, 18.260556°E
*Mordellistenatarsata* DSBS 39	OM680971	Slovakia, Virt env.	47.760000°N, 18.340556°E
*Mordellistenatarsata* DSBS 41	OM680972	Slovakia, Virt env.	47.760000°N, 18.340556°E
*Mordellistenatarsata* DSBS 42	OM680973	Slovakia, Virt env.	47.760000°N, 18.340556°E
*Mordellistenatarsata* DSBS 209	OM681010	Cyprus, Skoulli env.	34.968056°N, 32.446111°E
*Mordellistenatarsata* DSBS 210	OM681011	Bulgaria, Melnik env.	41.510000°N, 23.378333°E
*Mordellistenatarsata* DSBS 211	OM681012	Bulgaria, Melnik env.	41.510000°N, 23.378333°E
*Mordellochroaabdominalis* DSBS 138	OM681022	Slovakia, Burda	47.847778°N, 18.789722°E

The specimens selected for DNA isolation were killed in 96.3% ethanol and stored at -20 °C. The remaining specimens were killed with the fumes of ethyl acetate. After DNA isolation, the specimens were soaked in 5% acetic acid, dissected, and mounted on cards. The dissected genitalia were cleared in lactic acid for several days, or in 10% KOH overnight, then dehydrated in 96.3% ethanol and mounted on slides in Euparal (Paradox Co., Cracow, Poland). After examination, genitalia were mounted on the card with the respective specimen using dimethyl hydantoin formaldehyde (Entomopraxis, Barcelona, Spain). Specimens were observed under an MZ16 stereomicroscope (Leica, Wetzlar, Germany) with magnification up to 120× with diffused LED light (6400 K). The drawings were prepared using a drawing tube attached to a DM1000 compound microscope (Leica, Wetzlar, Germany) and subsequently inked with the Isograph technical pens (Rotring, Hamburg, Germany). Photographs of habitus were taken with an EOS 5D mark II camera (Canon, Tokyo, Japan) attached to an Axio Zoom V16 stereoscope (Zeiss, Oberkochen, Germany); photographs of genitalia were taken with an Axio Imager 2 (Zeiss, Oberkochen, Germany). The images were stacked in Zerene Stacker 1.4 software (https://zerenesystems.com/cms/stacker) and edited in Adobe Photoshop CC (https://www.adobe.com/products/photoshop.html) and DxO Photolab 5 (https://www.dxo.com/dxo-photolab/). For scanning electron microscopy, the body parts were disarticulated, cleaned in lactic acid for several days, dehydrated in 96.3% ethanol, coated with 25 nm thick gold layer, and examined using Quanta 250 FEG (FEI Europe B.V., Eindhoven, The Netherlands). Measurements were made with an ocular micrometre in a MZ16 stereomicroscope (Leica, Wetzlar, Germany) and are given in the text as the range followed by the arithmetic mean and standard deviation enclosed in parentheses. The measured characters are abbreviated in the text as follows:

**EL** elytral length from scutellar apex to elytral apices along suture;

**EW** maximum elytral width;

**HL** head length from anterior clypeal margin to occipital carina along midline;

**HW** maximum head width;

**LPrL** maximum left paramere length;

**PL** pronotal length along midline;

**PW** maximum pronotal width;

**PygL** maximum pygidial length;

**RPrL** maximum right paramere length;

**TL** combination of head, pronotal and elytral lengths.

The species description follows the conventional terminology as used in Franciscolo (1957), Lu et al. (1997), and Lawrence and Ślipiński (2010). Terminology for sensilla types follows Snodgrass (1935). All nomenclatorial acts follow the regulations of the International Code of Zoological Nomenclature (International Trust of Zoological Nomenclature 1999).

In total, 56 specimens were used for DNA isolation. Details on the voucher specimens, including sampling localities and GenBank accession numbers, are presented in Table 1. DNA was isolated from the entire specimens using the E.Z.N.A. Tissue DNA kit (OMEGA Biotek Inc., Norcross, GA, USA) according to the manufacturer’s protocol. The COI gene was amplified by PCR using standard primers LCO1490 and HCO2198 (Folmer et al. 1994). Individual PCR reactions were carried out in a total volume of 20 μl and included 10 μl of GoTaq Green master mix (Promega, Fitchburg, WI, USA), 0.52 μl of each primer (10 pmol/μl), 5 μl of extracted DNA, and 3.96 μl of nuclease-free water. The PCR thermocycler program was as follows: 94 °C for 120 sec, 40 cycles of 94 °C for 40 sec, 52 °C for 40 sec, 72 °C for 60 sec, and 72 °C for 10 min. PCR products were purified using EPPiC Fast (A&A Biotechnology, Gdansk, Poland) and sequenced from both sides in Macrogen Europe B.V. (Amsterdam, The Netherlands).

The consensus sequences, alignment, and final matrix were produced in Unipro UGENE 44.0 software (http://ugene.net/). Pairwise *p*-distances were calculated using MEGA X (Kumar et al. 2018). Maximum likelihood analysis (ML) was performed using IQ-TREE (Nguyen et al. 2015) on the web server (http://iqtree.cibiv.univie.ac.at) with the best substitution model (TIM2+F+I+G4) identified by the in-built ModelFinder according to BIC criterion. The node support values were obtained from 10,000 ultrafast bootstrap replicates (Hoang et al. 2017) and tested by the SH-aLRT branch test (Guindon et al. 2010). Bayesian inference was carried out in MrBayes 3.2.7a (Ronquist et al. 2012) on XSEDE available in CIPRES Science Gateway (https://www.phylo.org/index.php/). The number of substitution types was set to six, the nucleotide substitution model set to 4 × 4, and the among-site variation rate set to invgamma. Markov Chain Monte Carlo (MCMC) simulations included two independent runs each with four simultaneous chains, ten million generations, a sampling frequency of trees and parameters set to 1,000, and a burn-in fraction of 25%. The convergence of the MCMC analyses and an adequate sample size from the posterior distribution were confirmed using the in-built MrBayes diagnostics. Both trees were rooted subsequently in FigTree 1.4 (http://tree.bio.ed.ac.uk/software/figtree/), with *Mordellochroaabdominalis* (Fabricius, 1775) selected as outgroup. A distance-based species delimitation analysis, Assemble Species by Automatic Partitioning (ASAP) (Puillandre et al. 2020), was run on the ASAP web server (https://bioinfo.mnhn.fr/abi/public/asap/) using uncorrected *p*-distances. A tree-based species delimitation analysis, Poisson Tree Processes (PTP) (Zhang et al. 2013), was run on the bPTP web server (https://species.h-its.org) with the ML tree used as an input, in 100,000 MCMC generations with 25% burn-in fraction. The distribution of intraspecific, interspecific, and intergeneric distances was calculated from uncorrected *p*-distances using an Automatic Barcode Gap Discovery (ABGD) analysis (Puillandre et al. 2012) carried out on the ABGD web interface (https://bioinfo.mnhn.fr/abi/public/abgd/) in 20 steps, with P_min_ = 0.001, P_max_ = 0.1, and relative gap width = 1.5. Multidimensional scaling (MDS) of uncorrected *p*-distances was performed in IBM SPSS Statistics software (https://www.ibm.com/products/spss-statistics).

## ﻿Results

### ﻿COI gene analyses

**Figure 2.**
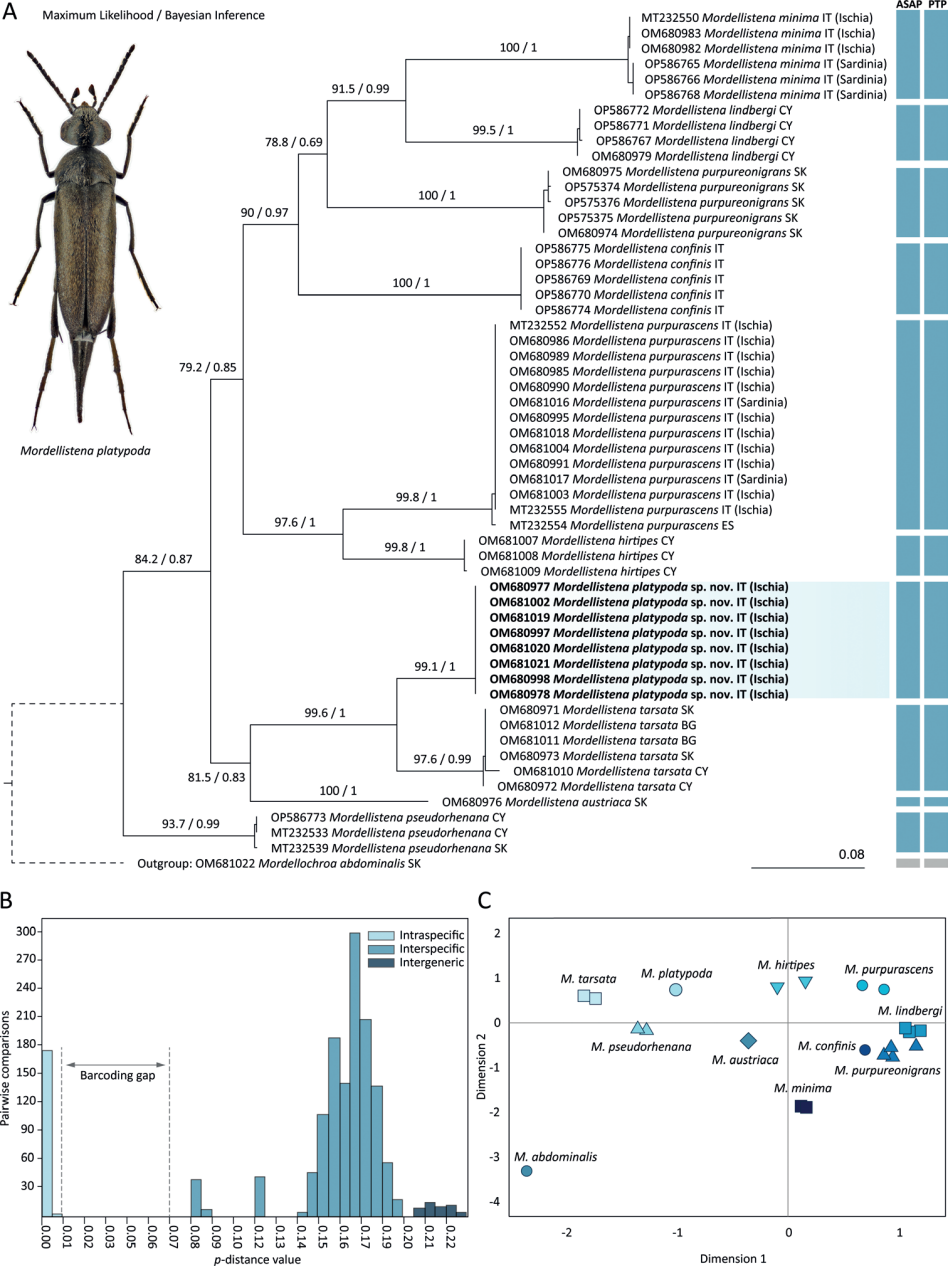
Analyses of a 658 bp fragment of the cytochrome c oxidase subunit I gene (COI) in selected *Mordellistena* A. Costa, 1854 species **A** combined results of maximum likelihood (ML) and Bayesian inference (BI) analyses; ML bootstrap values and BI posterior probabilities are shown on ML tree. The vertical bars represent results of Assemble Species by Automatic Partitioning (ASAP) and Poisson Tree Processes (PTP) species delimitation analyses **B** distribution of uncorrected *p*-distances **C** multidimensional scaling of uncorrected *p*-distances.

We generated and analysed a set of 56 sequences of the 658 bp COI gene fragment. The dataset comprised 237 parsimony-informative sites (36%) and 22 singleton sites (3.34%). We recovered almost identical topologies with both maximum likelihood (ML) and Bayesian inference (BI) methods, and therefore only the ML tree is presented with ML bootstrap values and BI posterior probability values (Fig. 2). ML and BI analyses revealed ten well-separated monophyletic clades (excluding outgroup) with high bootstrap (> 93) and posterior probability (> 0.99) values. Each of these clades represents a single species, initially recognised based on morphological characters. *Mordellistenaplatypoda* sp. nov. and *M.tarsata* Mulsant, 1856 were grouped in one clade with high statistical support (99.6/1.00). Another well-supported clade with high statistical support (97.6/1.00) consists of *M.purpurascens* and *M.hirtipes*. The *M.micans* species group which is defined by combination of morphological characters (represented here by *M.austriaca*, *M.hirtipes*, *M.minima* A. Costa, 1854, *M.platypoda*, *M.pseudorhenana* Ermisch, 1977, and *M.purpurascens*) was recovered as polyphyletic, with members of the *M.tarsata* species group (*M.tarsata*) and the *M.pumila* species group (*M.purpureonigrans*) placed within. ASAP and PTP delimitation methods recovered ten species (excluding outgroup) in congruence with morphology-based delimitation, ML, and BI analyses (Fig. 2). The lowest ASAP score was 1.5.

The uncorrected *p*-distances within and between species are summarised in Appendix 1. The lowest interspecific distance within the entire data set is between *M.platypoda* sp. nov. (represented by a single haplotype) and *M.tarsata* with a range of 8.21% to 8.97%. The highest interspecific distance within the ingroup is observed between *M.lindbergi* and *M.tarsata* (19.15–19.76%). The highest intraspecific distance (1.06%) is observed in *M.tarsata*. The distribution of *p*-distances (Fig. 2) shows a distinct gap between the highest intraspecific distance (1.06%) and the lowest interspecific distance (8.21%). The multidimensional scaling of *p*-distances shows separate clusters for each species (Fig. 2).

## ﻿Taxonomy

### ﻿ Mordellistena (s. str.) platypoda

Taxon classificationAnimaliaColeopteraMordellidae

Selnekovič, Goffová & Kodada sp. nov.

993DF123-594F-5DAC-B2C6-ECEA90EF7503

https://zoobank.org/A04D9CDE-AD8B-4DC0-966D-6B72082BE9AE

Figs 1 -7

#### Type locality.

Italy, Ischia, Serrara env., 40.72138°N, 13.88305°E; steep slopes with grassland communities, ca. 550 m alt. (Fig. 7).

#### Type material.

***Holotype***: italy • male; Ischia, Serrara env.; 40.72138°N, 13.88305°E; ca. 550 m alt.; 30 Jun 2019; D. Selnekovič leg.; steep slopes with grassland communities, on inflorescences of Apiaceae; GenBank: OM680978; SNSD. ***Paratypes***: italy • 9 males, 8 females; same data as for holotype • 23 males, 11 females; Ischia, Serrara env.; 40.71666°N, 13.88638°E; 517 m alt.; 29 Jun 2019; D. Selnekovič leg.; ruderal habitat on road verge, on inflorescences of *Daucuscarota*; GenBank: OM680977, OM680997, OM680998, OM681002, OM681019 to OM681021; SNSD.

#### Differential diagnosis.

**Figure 3.**
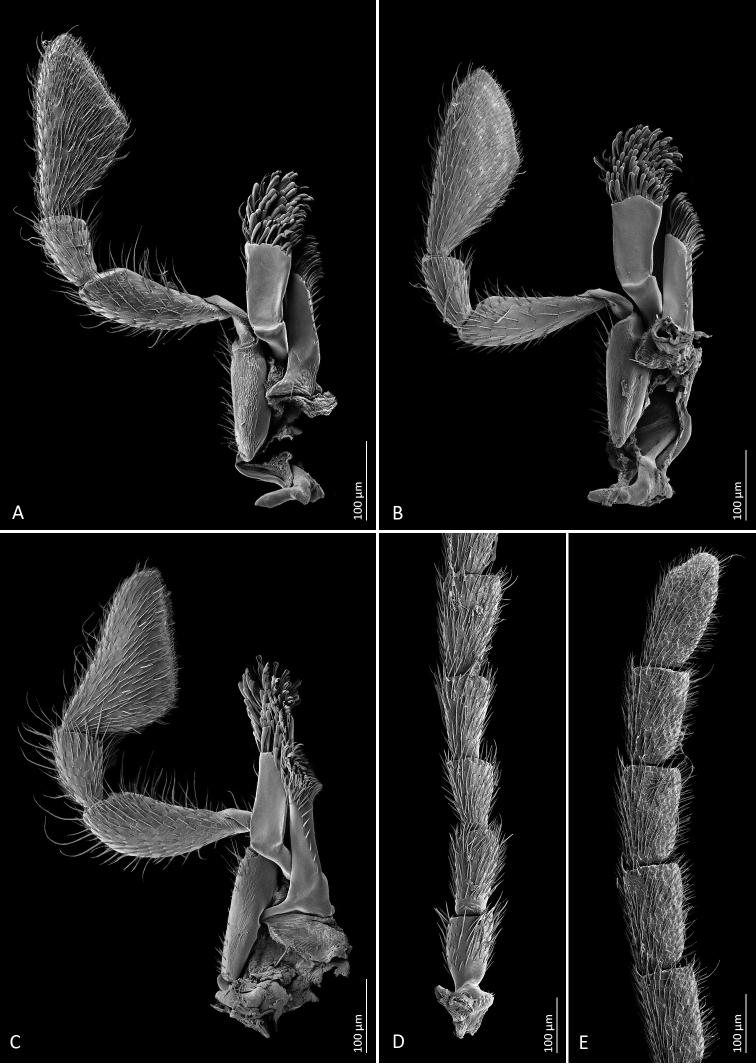
Scanning electron microscope images of diagnostic characters **A***Mordellistenaplatypoda* Selnekovič, Goffová & Kodada, sp. nov., male maxilla **B***M.platypoda*, female maxilla **C***M.purpurascens* A. Costa, 1854, male maxilla **D***M.platypoda*, male antenna (basal part) **E***M.platypoda*, male antenna (apical part).

**Figure 4.**
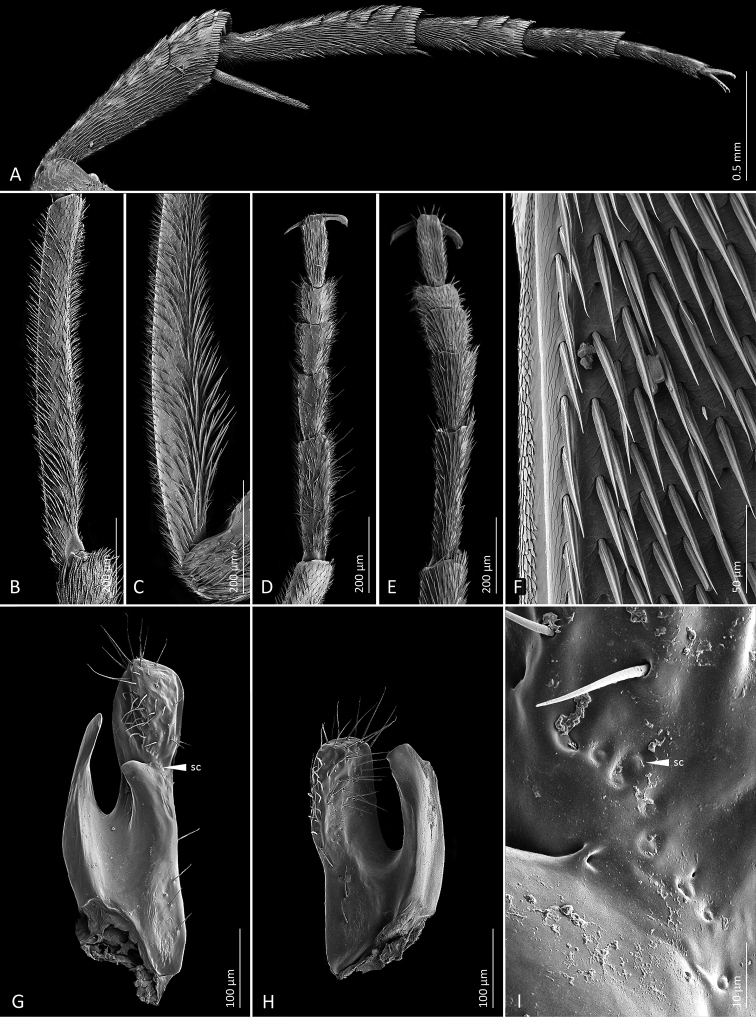
Scanning electron microscope images of diagnostic characters **A***Mordellistenaplatypoda* Selnekovič, Goffová & Kodada, sp. nov., male hind leg, lateral view **B***M.platypoda*, male protibia **C***M.purpurascens* A. Costa, 1854, male protibia **D***M.platypoda*, male protarsus **E***M.platypoda*, female protarsus **F***M.platypoda*, mesal portion of male right elytron **G***M.platypoda*, left paramere, mesal view, white triangle points to a cluster of sensilla campaniformia shown in image **I**; **H***M.platypoda*, right paramere, mesal view **I***M.platypoda*, cluster of sensilla campaniformia on left paramere. Abbreviation: sc = sensillum campaniformium.

**Figure 5.**
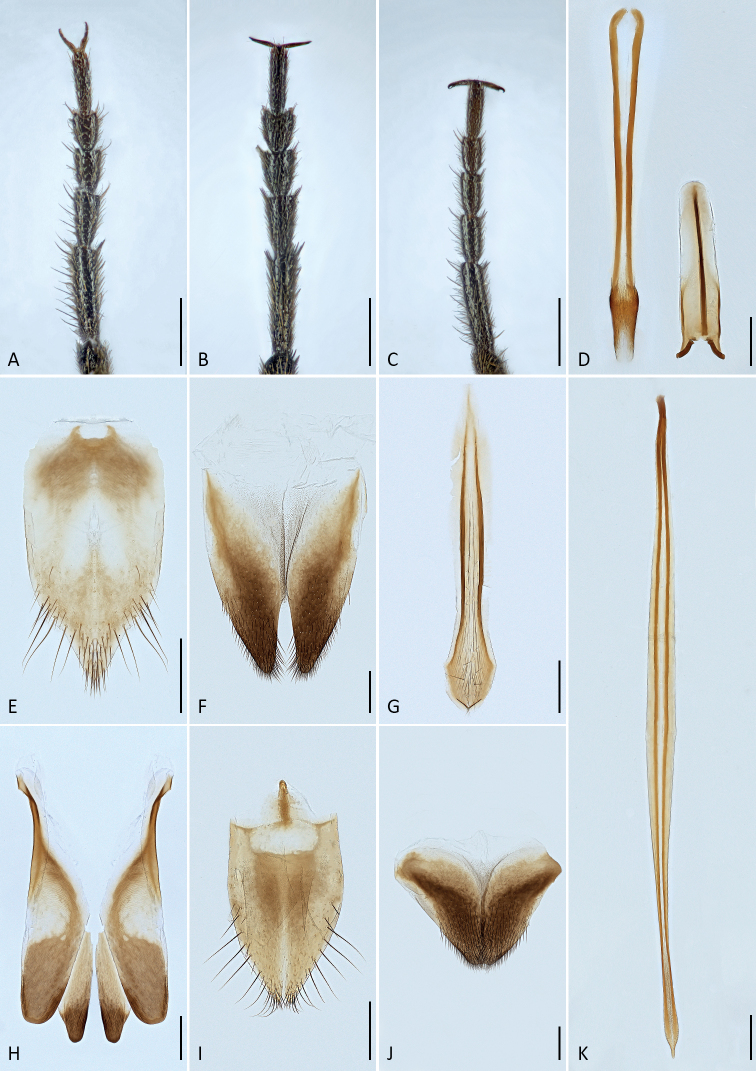
**A***Mordellistenaplatypoda* Selnekovič, Goffová & Kodada, sp. nov., male protarsus **B***M.platypoda*, female protarsus **C***M.purpurascens* A. Costa, 1854, male protarsus **D***M.platypoda*, phallobase **E***M.platypoda* male sternite VIII **F***M.platypoda*, male tergite VIII **G***M.platypoda*, male sternite IX **H***M.platypoda*, male tergites IX and X **I***M.platypoda*, female sternite VIII **J***M.platypoda*, female tergite VIII **K***M.platypoda*, penis. Scale bars: 0.1 mm.

*Mordellistenaplatypoda* is included in the *M.micans* species group as defined by Batten (1977) on the basis of the following characters: the four first antennomeres are narrower and shorter than the following ones (Fig. 3D); hind tibia besides the subapical ctenidium with four lateral ctenidia that are more or less perpendicular to the dorsal edge of the tibia (Fig. 4A); only the first two metatarsomeres with lateral ctenidia (Fig. 4A); punctation of elytra not conspicuously coarse; metatibial spurs black. A rather unique character that separates the species from most of its congeners is the shape of pro- and mesotarsomeres, which are flattened and expanded. The entire male mesotarsus and the female pro- and mesotarsus are dilated apically (Figs 4D, E, 5A, B). Similarly formed tarsi are present in *M.rugipennis* Schilsky, 1895 and *M.latitarsis* Batten, 1983, both of which differ from *M.platypoda* in the entirely black vestiture and shape of the genitalia. Furthermore, *M.platypoda* is characterised by its large body dimensions (TL: ♂♂ 4.58–5.64 mm, ♀♀ 4.84–6.02 mm). Similarly large body is present within the *M.micans* species group only in *M.purpurascens* A. Costa, 1854 (TL: ♂♂ 4.27–5.76 mm, ♀♀ 4.32–5.78 mm), *M.hirtipes* Schilsky, 1895 (TL: ♂♂ 4.13–5.16 mm, ♀♀ 3.69–4.77 mm), and *M.austriaca* Schilsky, 1898 (TL: ♂♂ 3.85–5.37 mm, ♀♀ 3.67–5.33 mm). The latter species is separated from *M.platypoda* by almost square antennomeres 5–10, the lateral pronotal sides straight in the lateral aspect, the posterolateral pronotal angels obtuse, the elytra shorter (EL/EW ratio ≤ 2.2), and the genitalia differently shaped.

*Mordellistenaplatypoda* most closely resembles a sympatric species *M.purpurascens* but differs in paler vestiture, weakly expanded male second maxillary palpomere, with few long setae (Fig. 3A) compared to strongly expanded, with numerous long setae in *M.purpurascens* (Fig. 3C), weakly expanded male protibia, with few inconspicuous extended setae (Fig. 4B) compared to strongly expanded, with conspicuous long setae in *M.purpurascens* (Fig. 4C), protarsus expanded in both sexes and distinctly dilated apically in females (Fig. 5A, B) compared to weakly narrowed apically in *M.purpurascens* (Fig. 5C), elytra longer, with EL/EW ratio: ♂♂ 2.31–2.64, ♀♀ 2.22–2.40 compared to ♂♂ 1.97–2.23, ♀♀ 1.83–2.15 in *M.purpurascens*, and parameres distinctly shorter and differently shaped (Figs 4G, H, 5B; EL/LPrL ratio: 7.09–8.59, EL/RPrL ratio: 9.67–12.08) than in *M.purpurascens* (EL/LPrL ratio: 4.42–5.84, EL/RPrL ratio: 5.57–6.94; see Selnekovič and Kodada 2019: fig. 7). *Mordellistenatenuicornis* Schilsky, 1899 is separated from *M.platypoda* by the presence of two or three lateral ctenidia on the third metatarsomere, antennomeres 5–10 ca. 2.0× longer than wide, and pygidium distinctly longer and more slender.

The results of the COI gene analyses show the close relationship of *M.platypoda* with *M.tarsata* Mulsant, 1856, with *p*-distances of 8.21–8.97%. The two species are easily distinguished by the colouration of the dorsal vestiture, which is black with a greenish lustre in *M.tarsata* compared to yellowish in *M.platypoda* (Fig. 1), the presence of two lateral ctenidia on the third metatarsomere in *M.tarsata* compared to the absence of lateral ctenidia on the segment in *M.platypoda*, the presence of a conspicuous cluster of long setae in the proximal portion of the male protibia in *M.tarsata* compared to few elongated setae in in *M.platypoda*, and the different shape of the parameres.

#### Description.

Measurements (in mm; ♂♂ *n* = 33, ♀♀ *n* = 21): TL: ♂♂ 4.58–5.64 (5.25 ± 0.26), ♀♀ 4.84–6.02 (5.56 ± 0.31); HL: ♂♂ 0.81–1.01 (0.92 ± 0.05), ♀♀ 0.84–1.06 (0.97 ± 0.06); HW: ♂♂ 0.94–1.17 (1.07 ± 0.05), ♀♀ 0.95–1.19 (1.10 ± 0.06); PL: ♂♂ 0.98–1.25 (1.14 ± 0.06), ♀♀ 1.06–1.33 (1.22 ± 0.08); PW: ♂♂ 1.10–1.46 (1.32 ± 0.08), ♀♀ 1.19–1.57 (1.42 ± 0.10); EL: ♂♂ 2.75–3.50 (3.18 ± 0.18), ♀♀ 2.92–3.67 (3.37 ± 0.19); EW: ♂♂ 1.13–1.42 (1.31 ± 0.07), ♀♀ 1.25–1.54 (1.45 ± 0.08); RPrL: 0.27–0.32 (0.29–0.01); LPrL: 0.36–0.43 (0.40 ± 0.02).

Body elongated, wedge-shaped, widest in anterior half of elytra (Fig. 1). Dorsum slightly convex, venter strongly so. Entire body surface uniformly black, except for reddish brown anteclypeus. Vestiture consisting of decumbent lanceolate setae (Fig. 4F); yellowish, darkened before elytral apices and along posterior margin of ventrites 4 and 5.

Head large, transverse, moderately convex dorsally, with highest point behind middle of eyes (lateral aspect), HW/HL ratio: ♂♂ 1.09–1.23 (1.16 ± 0.03), ♀♀1.01–1.23 (1.13 ± 0.05); occipital carina convex; integument weakly microreticulate, weakly iridescent, with small round setiferous punctures. Eyes broadly oval, vertical diameter ca. 1.3× horizontal diameter; posteriorly reaching to occipital margin; finely faceted; interfacetal setae longer than facet diameter. Anterior clypeal edge weakly convex. Labrum transverse, densely setose, anterior edge weakly convex. Antenna weakly serrate (Fig. 3D, E); antennomeres 1–4 shorter and narrower than following segments; scape cylindrical, little longer than wide; pedicel cylindrical, little longer than scape; antennomere 3 little longer than wide, expanded distally; antennomere 4 little longer than 3; antennomeres 5–10 in both sexes 1.6–1.7× as long as wide; antennomere 11 oval, ca. 2.2× as long as wide; all antennomeres covered with decumbent sensilla chaetica; antennomeres 5–10 each with several long and erect sensilla trichoidea apico-laterally (Fig. 3D, E). Galea short, with spatulate sensilla and sensilla chaetica apically (Fig. 3A, B). Lacinia with numerous sensilla chaetica apically and row of sensilla chaetica along mesal margin (Fig. 3A, B). Maxillary palpomere 2 subcylindrical, weakly expanded distally, in males little wider and with somewhat longer sensilla chaetica than in females (Fig. 3A, B); terminal maxillary palpomere scalene triangular, mesal angle in middle or in anterior half, numerous decumbent sensilla chaetica and several erect sensilla chaetica over entire surface, several sensilla placoidea before distal margin.

**Figure 6.**
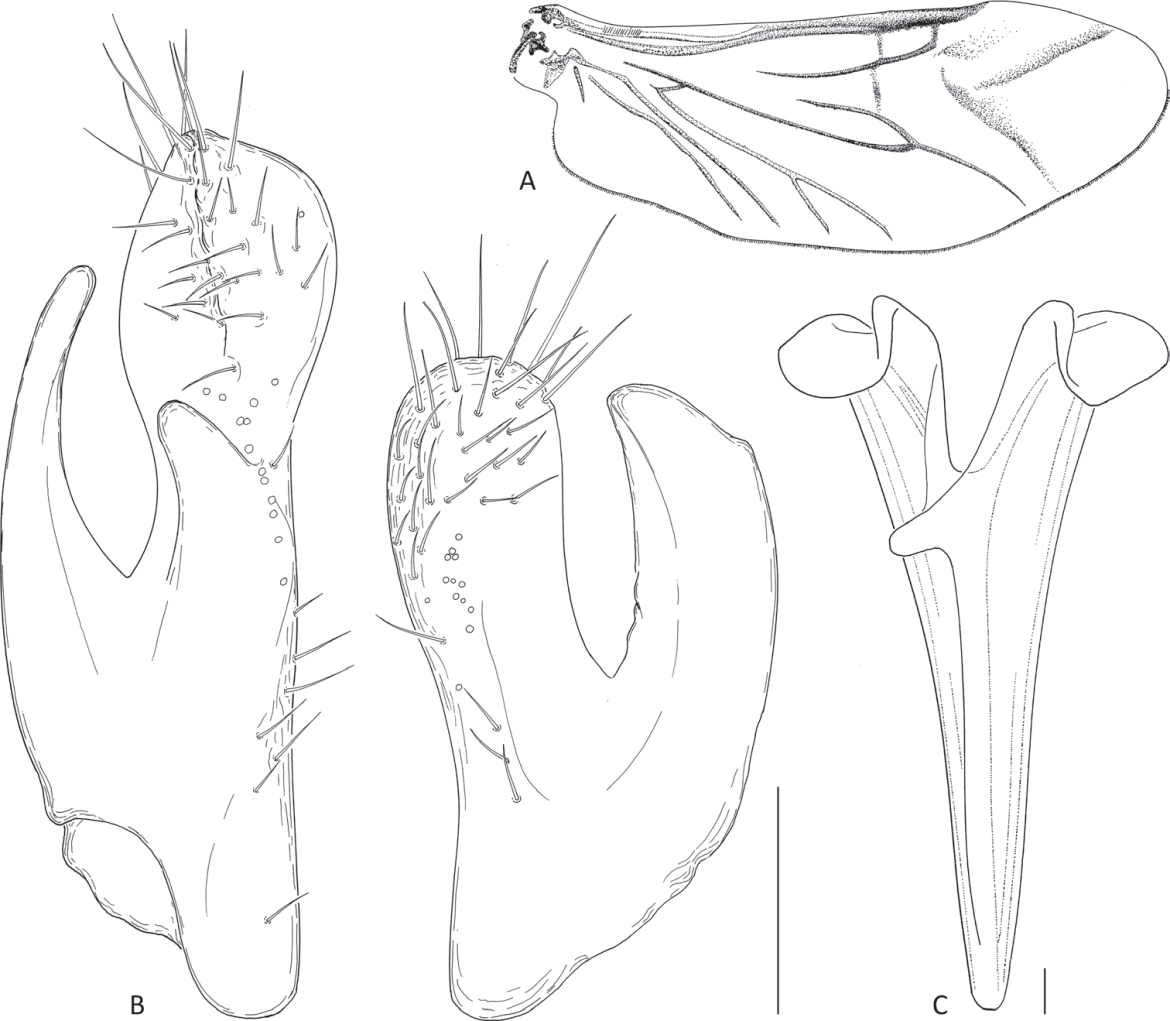
*Mordellistenaplatypoda* Selnekovič, Goffová & Kodada, sp. nov., paratype **A** male right hindwing **B** parameres **C** metendosternite. Scale bars: 0.1 mm.

Pronotum 1.1–1.2× as wide as long, widest behind middle, moderately convex; surface microreticulate, densely covered with lanceolate setae, punctures larger than those on head; anterior edge convex in middle, anterolateral angles broadly rounded; lateral carinae sinuate in lateral aspect; posterior edge sinuate, posterolateral angles rectangular in lateral aspect. Scutellar shield triangular, densely setose. Elytra widest between first and second quarter, EL/EW ratio: ♂♂ 2.31–2.64 (2.43 ± 0.07), ♀♀ 2.22–2.40 (2.32 ± 0.05); apices separately rounded; surface microreticulate, densely covered with decumbent lanceolate setae, punctures coarser than those on pronotum. Hindwing as in Fig. 6A. Mesoventral process about as wide as mesotibia. Metaventral discrimen apparent. Metanepisternum ca. 2.3× longer than greatest width, narrowed posteriorly. Metendosternite as in Fig. 6C. Protibia ca. 1.1× longer than protarsus; in males weakly expanded proximally and with few inconspicuous extended setae (Fig. 4B). Protarsus expanded and flat in both sexes, weakly dilated distally in females (Figs 4D, E, 5A, B); protarsomere 1 little shorter than three following tarsomeres combined; penultimate protarsomere weakly expanded distally, with apical edge concave; each protarsal claw with four denticles. Mesotibia ca. 0.8× as long as mesotarsus. Mesotarsus dilated distally in both sexes; first mesotarsomere nearly as long as three subsequent tarsomeres combined. Metatibia with short subapical ctenidium and four lateral ctenidia nearly perpendicular to dorsal tibial edge, proximal ctenidium often rudimentary (Fig. 4A); outer terminal spur ca. 0.75× as long as inner one. Metatarsomere 1 with four or five lateral ctenidia; metatarsomere 2 with two lateral ctenidia; metatarsomeres 3 and 4 without lateral ctenidia (Fig. 4A).

**Figure 7.**
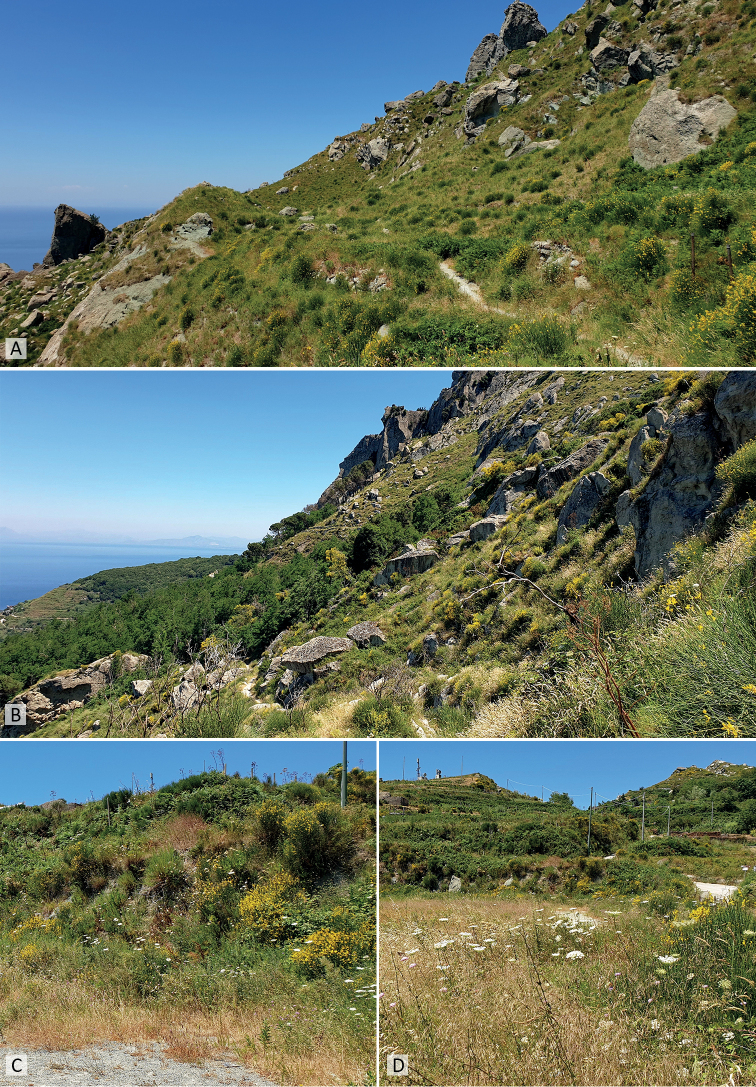
Habitats of *Mordellistenaplatypoda* Selnekovič, Goffová & Kodada, sp. nov. near Serrara village, Ischia, Italy **A, B** type locality, slopes with Mediterranean grassland communities (40.72138°N, 13.88305°E) **C, D** ruderal communities with *Daucuscarota* Linnaeus along road (40.71666°N, 13.88638°E).

Abdominal ventrite 1 longer than ventrite 2; ventrite 5 with arcuate apical edge. Pygidium long, conical, narrowly truncate at apex, EL/PygL ratio: ♂♂ 1.75–2.03 (1.87 ± 0.06), ♀♀ 2.12–2.35 (2.21 ± 0.06). Male tergite VIII deeply emarginate on posterior edge, setose apically (Fig. 6F); female tergite VIII divided by longitudinal suture basally (Fig. 5J), setose apically. Male sternite VIII strongly produced in middle of posterior edge, with long setae (Fig. 5E); female sternite VIII produced in middle of posterior edge, setose (Fig. 5H), anterior median strut short, narrowly elliptical. Male tergite IX completely divided into two parts, each with narrow basal projection (Fig. 5H). Male sternite IX narrow, strongly sclerotised at lateral edges, expanded before apex, with several sensilla trichoidea (Fig. 5G). Male tergite X divided into two parts, partly fused to tergite IX (Fig. 5H). Phallobase forming sheath around penis; tubular part short; anterior struts ca. 3.1× as long as tubular part; dorsal apodeme strongly sclerotised, lateral edges even (Fig. 5D). Parameres as in Figs 4G, H, 6B: left paramere longer than right one, EL/LPrL ratio: 7.09–8.59 (7.97 ± 0.41), dorsal process moderately dilated and obliquely truncate apically, with numerous sensilla trichoidea, ventral process shorter than dorsal one, slightly bent dorsad, narrowly rounded apically, median process short, produced ventrad, cluster of approximately nine sensilla campaniformia present above dorsal edge of median process (Figs 4I, 6B); left paramere with dorsal process subtruncate apically, with trichoid and campaniform sensilla, ventral process slightly shorter than dorsal one, bent dorsad, subtruncate at apex, EL/RPrL ratio: 9.67–12.08 (10.92 ± 0.58). Penis long, narrow, weakly expanded before apex (Fig. 5K). Ovipositor: proctiger moderately long, with sclerotised lateral baculi; paraprocts slightly shorter than gonocoxites, with sclerotised baculi; gonocoxites ventrally divided, setose, with oblique basal baculi; gonostyli attached subapically, each with two trichoid sensilla at apex.

#### Secondary sexual dimorphism.

Females are on average slightly larger than males. Males are more slender than females (Fig. 1). The second maxillary palpomere has longer setae in males than in females (Fig. 3A, B). Terminal maxillary palpomere is slightly narrower in females (Fig. 3A, B). The male protibia bears several elongate setae in proximal half (Fig. 4B), while the female protibia is uniformly setose. Male protarsomeres bear numerous thick setae oriented mesoventrad (Figs 4D, 5A). Protarsus and mesotarsus are more strongly dilated distally in females (Figs 4D, E, 5A, B).

#### DNA sequences.

Partial COI gene sequences of holotype and eight paratypes were submitted to GenBank (https://www.ncbi.nlm.nih.gov/genbank/). The accession numbers are listed in Table 1.

#### Etymology.

The specific epithet is derived from the Greek words *πλατύς* (platýs), meaning wide, broad, and *πόδι* (pódi) meaning foot. It refers to the expanded pro- and mesotarsi, an unusual condition that separates *M.platypoda* from morphologically similar congeners.

#### Distribution.

The species is known only from the island of Ischia in Italy.

#### Collecting notes and habitat.

*Mordellistenaplatypoda* was sampled in a series of 52 specimens on 29–30 June 2019. The sampling was carried out at two nearby localities (approximately 600 m apart) near Serrara village. The type locality (40.72138°N, 13.88305°E) was characterised by the steep rocky slopes with Mediterranean grassland communities (Fig. 7A, B). The specimens were collected by sweeping the inflorescences of Apiaceae spp. At the second location (40.71666°N, 13.88638°E), the specimens were collected from the inflorescences of *Daucuscarota* that grew in a ruderal community along the road (Fig. 7C, D). During the same collection events, the following Mordellidae species were also collected: *Mediimordabipunctata* (Germar, 1823), *Mordellaaculeata* Linnaeus, 1758, *Mordellistenaepisternalis* Mulsant, 1856, *M.hirtipes* Schilsky, 1895, *M.minima* A. Costa, 1854, *M.pseudorhenana* Ermisch, 1977, *M.purpurascens* A. Costa, 1854, *M.wiebesi* Batten, 1977, and *Variimordabasalis* (A. Costa, 1854).

## ﻿Discussion

*Mordellistenaplatypoda* is morphologically well defined. It is included in the *M.micans* species group defined by Batten (1977). It should be noted, however, that the results of the molecular analyses presented in this study as well as other preliminary and yet unpublished results show that, despite the similarity in morphological characters shared by its members, the *M.micans* species group is polyphyletic, and therefore we mention it only marginally in this study to facilitate species identification. The recognition of the new species and the species hypothesis were based primarily on the unique combination of morphological characteristics, e.g., remarkably large body, expanded and dilated pro- and mesotarsus, length of antennal segments, inconspicuous secondary sexual dimorphism in the protibia, and unique shape of the genitalia. Expanded and dilated fore and middle tarsi are present in both sexes, but the condition is more pronounced in females. It is an uncommon character state present, among the European species, only in *M.rugipennis* Schilsky, 1895 (Horák 1990) and *M.latitarsis* Batten, 1983. Both species differ from *M.platypoda* by the black vestiture pubescence on the dorsal surfaces. Another notable characteristic of *M.platypoda* is its large body that reaches up to 5.6 mm in males and 6.2 mm in females (TL). Comparably large species within the *M.micans* group are *M.purpurascens* A. Costa, 1854 and *M.hirtipes* Schilsky, 1895, which are sympatric with *M.platypoda*. Both species can be easily distinguished from *M.platypoda* by different shapes of the protarsus and mesotarsus, shorter antennal segments, distinct sexual dimorphism in the protibia, and different shapes of genitalia (Selnekovič and Kodada 2019).

The morphology-based species hypothesis was evaluated by analysing a 658 bp fragment of the COI gene. This standard DNA barcoding marker has frequently been used in the taxonomy of beetles not only to identify species as originally proposed (Hebert et al. 2003; DeSalle and Goldstein 2019), but also to explore species boundaries and refine morphology-based hypotheses (Pentinsaari et al. 2016; Fossen et al. 2016; Bergsten et al. 2017; Maddison and Sproul 2020). Despite the frequent use of this marker in beetle taxonomy, it has only been used once in the study of Mordellidae to identify species and interpret morphological variability (Selnekovič et al. 2021). Here, we recovered a phylogenetic tree using two probabilistic methods, maximum likelihood (ML) and Bayesian inference (BI). To assess the accuracy of the results, we compared them with those of distance-based (ASAP) and tree-based (PTP) species delimitation methods. ML and BI analyses show well-separated homogenous clades with strong statistical support for each presumed species including *M.platypoda* (ML bootstrap values higher than 97 and BI posterior probabilities 1.00) (Fig. 2). *Mordellistenaplatypoda* forms a clade with *M.tarsata* Mulsant, 1856 with strong statistical support (ML 97, BI 1.00). A close relationship between the two species is also supported by the interspecific divergence of 8.21%–8.87%, which is the lowest within the entire data set. *Mordellistenaplatypoda* and *M.tarsata* also have several morphological similarities, e.g., overall body shape, length of antennal segments, and shape of genitalia. However, despite the close genetic relatedness, *M.platypoda* and *M.tarsata* can be distinguished easily by the different colouration of the dorsal vestiture, which is black in *M.tarsata* and yellowish in *M.platypoda*, and by the absence of lateral ctenidia on the third metatarsomere in *M.platypoda*. Such characters were traditionally used to differentiate species groups among European representatives of the genus *Mordellistena*, for example, the *M.micans* group vs. *M.tarsata* and *M.pumila* group. Interestingly, our results show that such differences may, in fact, also be present among closely related species. It should be mentioned that our molecular analyses did not include species such as *M.tenuicornis*, *M.latitarsis*, and *M.rugipennis*, which share several morphological similarities with *M.platypoda*; therefore, future studies may provide new insights into the phylogenetic relationships between the aforementioned species.

The distribution of pairwise genetic distances (*p*-distances) shows a distinct gap between the highest intraspecific divergence (1.06%) and the lowest interspecific divergence (8.21%) (Fig. 2). Genetic distances between the analysed *Mordellistena* species range from 8.21% to 19.76%, similar to the previous analyses (Selnekovič et al. 2021). The multidimensional scaling of the *p*-distances shows separate clusters for each species (Fig. 2).

*Mordellistenaplatypoda* was collected in a relatively large series of 52 specimens during one collecting event on the island of Ischia in Italy. Revision of the museum specimens identified as *M.micans* (Germar, 1817), *M.stenidea* Mulsant, 1856, and *M.grisea* Mulsant, 1856 in the Franciscolo collection in MSNG, a series of *M.micans* in SDEI, and a series of *M.micans* in HNHM did not reveal any additional specimens of the new species. Therefore, *M.platypoda* can now be considered endemic to the island of Ischia. However, given the island’s proximity to the shore (ca. 30 km) and the similarity of its fauna to that of the mainland, it is possible that *M.platypoda* may also occur on the Italian mainland or surrounding islands.

## Supplementary Material

XML Treatment for Mordellistena (s. str.) platypoda

## Appendix 1

**Table A1.** Uncorrected *p*-distances between and within the analysed species calculated in Mega X software. Intraspecific divergences are highlighted in bold.

	*M.austriaca*	*M.confinis*	*M.hirtipes*	*M.lindbergi*	*M.minima*	*M.platypoda*	*M.pseudorhenana*	*M.purpurascens*	*M.purpureonigrans*	*M.tarsata*	*M.abdominalis*
*Mordellistenaaustriaca*	**N/A**										
*M.confinis*	0.1505	**0.0000**									
*M.hirtipes*	0.1581–0.1596	0.1581–0.1596	**0.0000–0.0015**								
*M.lindbergi*	0.1672–0.1687	0.1702–0.1733	0.1550–0.1581	**0.0000–0.0030**							
*M.minima*	0.1565–0.1596	0.1581–0.1596	0.1884–0.1915	0.1474–0.1505	**0.0000–0.0046**						
*M.platypoda*	0.1489	0.1687	0.1535–0.1550	0.1717–0.1733	0.1763–0.1793	**0.0000**					
*M.pseudorhenana*	0.1565	0.1839	0.1626–0.1672	0.1702–0.1748	0.1733–0.1748	0.1611	**0.0000–0.0030**				
*M.purpurascens*	0.1641–0.1657	0.1581–0.1596	0.1170–0.1201	0.1657–0.1687	0.1793–0.1824	0.1672–0.1687	0.1733–0.1748	**0.0000–0.0046**			
*M.purpureonigrans*	0.1489–0.1535	0.1626–0.1672	0.1505–0.1550	0.1626–0.1702	0.1641–0.1687	0.1778–0.1824	0.1702–0.1733	0.1535–0.1581	**0.0000–0.0046**		
*M.tarsata*	0.1398–0.1474	0.1824–0.1900	0.1657–0.1702	0.1915–0.1976	0.1854–0.1945	0.0821–0.0897	0.1596–0.1657	0.1763–0.1793	0.1809–0.1869	**0.0000–0.0106**	
*M.abdominalis*	0.1991	0.2112	0.2158–0.2173	0.2295–0.2325	0.2036	0.2158	0.2036	0.2264–0.2280	0.2112–0.2143	0.2112–0.2128	**N/A**
